# Force Mapping Study of Actinoporin Effect in Membranes Presenting Phase Domains

**DOI:** 10.3390/toxins13090669

**Published:** 2021-09-18

**Authors:** Katia Cosentino, Edward Hermann, Nicolai von Kügelgen, Joseph D. Unsay, Uris Ros, Ana J. García-Sáez

**Affiliations:** Interfaculty Institute of Biochemistry, University of Tübingen, 72076 Tübingen, Germany; kacosentino@uos.de (K.C.); edumir930@googlemail.com (E.H.); Nicolai.vonKuegelgen@mdc-berlin.de (N.v.K.); joe.unsay@gmail.com (J.D.U.); uris.ros@uni-koeln.de (U.R.)

**Keywords:** pore forming toxins, atomic force microscopy, force spectroscopy, membrane phase domains

## Abstract

Equinatoxin II (EqtII) and Fragaceatoxin C (FraC) are pore-forming toxins (PFTs) from the actinoporin family that have enhanced membrane affinity in the presence of sphingomyelin (SM) and phase coexistence in the membrane. However, little is known about the effect of these proteins on the nanoscopic properties of membrane domains. Here, we used combined confocal microscopy and force mapping by atomic force microscopy to study the effect of EqtII and FraC on the organization of phase-separated phosphatidylcholine/SM/cholesterol membranes. To this aim, we developed a fast, high-throughput processing tool to correlate structural and nano-mechanical information from force mapping. We found that both proteins changed the lipid domain shape. Strikingly, they induced a reduction in the domain area and circularity, suggesting a decrease in the line tension due to a lipid phase height mismatch, which correlated with proteins binding to the domain interfaces. Moreover, force mapping suggested that the proteins affected the mechanical properties at the edge, but not in the bulk, of the domains. This effect could not be revealed by ensemble force spectroscopy measurements supporting the suitability of force mapping to study local membrane topographical and mechanical alterations by membranotropic proteins.

## 1. Introduction

Pore-forming toxins (PFTs) are involved in the defence system of many organisms, ranging from bacteria to animals. They compromise the integrity and functionality of target cells by permeabilization of the plasma membrane through pore formation [1,2,3]. Equinatoxin II (EqtII) and Fragaceatoxin C (FraC) are PFTs from the family of actinoporins, isolated from the sea anemones *Actina equine* and *Actinia fragacea*, respectively [4]. These toxins consist of a β-sandwich core skirted by two α-helices on opposite sides [5]. Their mechanism of action involves binding to the target membrane, a conformational change that facilitates the insertion of the N-terminal α-helix into the lipid bilayer [6], oligomerization and pore formation [7]. Evidence by single-molecule approaches have revealed that the number of monomeric units composing EqtII oligomers is variable [8], having a broad stoichiometry distribution [8,9]. This variability is in agreement with a model in which these α-PFTs form toroidal pores where both proteins and lipids contribute to the channel architecture [7,10]. The resolved crystal structure of the pore formed by FraC shows the presence of lipids between two adjacent proteins in order to stabilize the oligomeric complex formation [11]. The pore-forming activity of actinoporins is enhanced by the presence of sphingomyelin (SM) in the target membrane [4,10,12]. It has been suggested that this lipid can act as a receptor for these toxins, enhancing their binding through interaction with multiple binding sites [10,11]. This is in line with observations in artificial and cellular systems showing that membrane re-organization, favouring the formation of lipid domains in which SM is likely exposed to the domain boundaries, plays a role in promoting actinoporin-binding and activity [13,14,15]. It has been proposed that sticholysins, also members of the actinoporin family, induce the mixing of lipids from different membrane domains [16]. However, how membrane phase separation helps the action of actinoporins remains unclear [17,18,19,20], and even less is known about the changes that actinoporins induce in the membrane in order to permeabilize it.

Here, we investigated the effect of EqtII and FraC on membrane models having SM/Cholesterol (Chol) liquid ordered (Lo) domains dispersed in a 1,2-Dioleoyl-sn-glycero-3-phosphocholine (DOPC) liquid disordered (Ld) phase. In particular, we studied the changes induced by these actinoporins on the membrane topology and physical properties by using a combination of confocal microscopy and force mapping by atomic force microscopy (AFM).

**Figure 1:**
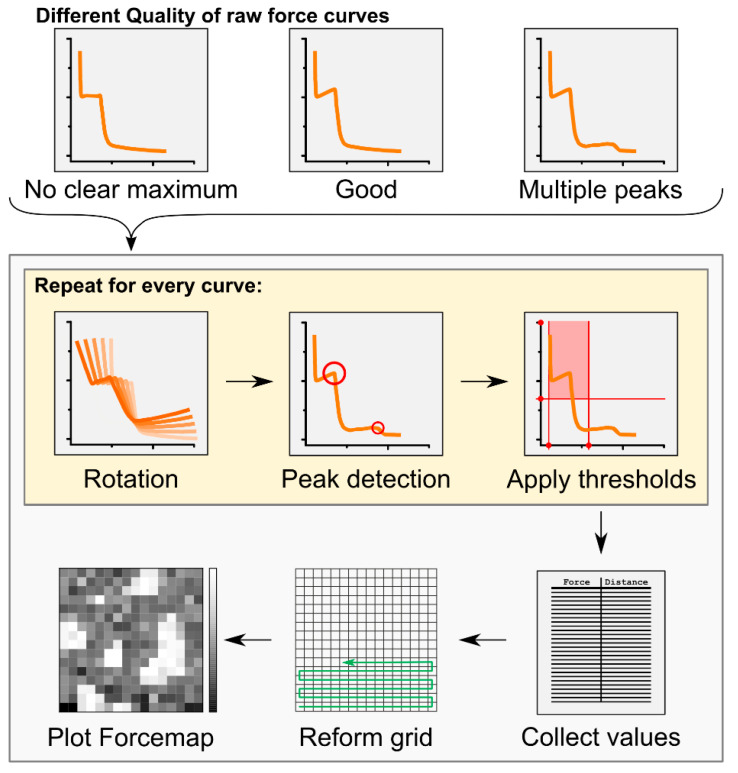
Workflow of the Forcemap Analyser program. Detected force curves obtained by the force mapping tool of the AFM were processed by the Forcemap Analyser program to obtain a spatially reconstructed forcemap of the sample. For details see “Automatic force map analysis” in the Methods and Materials section.

Force mapping has already been successfully applied to the study of membranes presenting Lo phase domains [21,22,23,24,25,26]. It offers the unique advantage of combining topographical and mechanical information from AFM, providing local information at the nanoscopic level that could not be revealed by the independent use of single imaging and force spectroscopy techniques. Its use in the past has often been limited by the long processing time of the huge number of collected force curves. Here, we developed an automatic analysis tool that allows for high-throughput calculations of breakthrough force and thickness values from force mapping data (Figure 1, Table 1, see also the Materials and Methods section). Although self-developed algorithms providing an automatic analysis of force mapping data have already been presented [25,26], our tool allows a faster analysis of the data because it is more user-friendly. We provide this as a Python executable file with a graphic user interface that can be universally applied to any kind of force mapping data (available in Github). 

In our experiments, force mapping data provide a valuable complement to the fluorescence imaging information, showing a strong rearrangement of the Lo domains and a reduction in the area and circularity of the domains in the presence of actinoporins. Importantly, the use of force mapping indicates that the proteins do not induce significant variations of the membrane properties in the bulk of neither the Lo nor the Ld phase, suggesting that the action of these proteins is mainly at the domain boundaries. These data are in agreement with findings suggesting the preferential binding of these proteins to the domain interface [17]. Our approach provides, in very short timescales, relevant information on the local effect of actinoporins in membranes presenting phase domains, and confirms force mapping as a powerful unprecedented tool to get nanoscopic-resolved information on mechanical properties of lipid membranes.

## 2. Results

### 2.1. Automatic Force Mapping Allows for a Fast-Spatial Characterization of Membrane Properties

**Figure 2:**
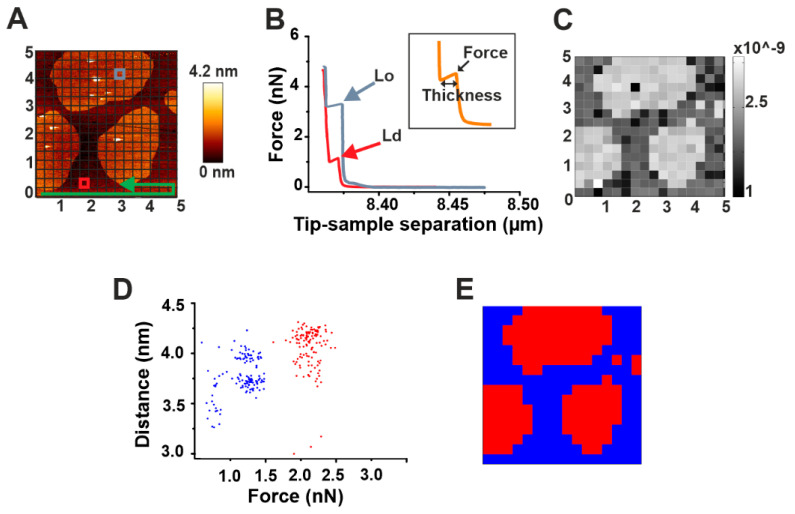
Automatic force mapping analysis. (**A**) Representative AFM figure of a DOPC/SM/Chol 2:2:1 supported lipid bilayer (SLB). The brighter round areas represent Lo domains whose thickness exceeds the surrounding Ld phase (darker area). In the Figure, the principle of force mapping is illustrated: the imaged area is divided by a grid in small sub-areas. For each of these sub-areas, following the direction indicated by the green arrow, a force spectroscopy measurement is performed. (**B**) Representative force spectroscopy curves for Lo (gray) and Ld (red) phases measured in correspondence of the gray and red squares, respectively, in (**A**). The inset depicts the force and thickness values, as measured for each curve with our automatic force mapping tool. (**C**) The topographical picture of Figure A could be reproduced nicely by our software by spatially plotting the force values. (**D**) Dot plot of all corresponding force and distance values, coloured according to their affiliation with one cluster determined by the KMeans algorithm. (**E**) Map of the same 5 μm × 5 μm area with each spot colored according to its affiliation with one cluster determined by the KMeans algorithm.

Force mapping is a useful tool for a direct spatial correlation of topographical and nano-mechanical properties of membranes. It exploits the force spectroscopy tool of AFM, which allows for getting information on both the force required to break the bilayer and its thickness [27]. A selected area of the sample, after imaging, is divided by a grid in small sub-areas (Figure 2A). For each of these sub-areas, a force spectroscopy measurement is performed (Figure 2B). Upon contact of the AFM tip with the sample, the pressure that is exerted on it induces a continuous increase until it reaches a threshold value. At this point, a discontinuity in the curve is observed (peak) corresponding to the force required to break the membrane (breakthrough force). The length of such discontinuity measures the thickness of the bilayer (inset in Figure 2B). Successively, the image of the total area can be reconstructed by spatially plotting the force values.

Despite its clear advantage, this technique requires long processing times for the analysis, due to the high amount of data collected per experiment (approximately 2000 force curves in our case) and the lack of a fast analysis software, commonly not provided by the AFM manufacturer. Specifically, both (i) the detection of the force and thickness values for each force curve and (ii) the spatial correlation of the force spectroscopy data have to be processed manually, resulting in a time-consuming procedure.

To solve this problem, we have developed an automatic processing tool (see Materials and Methods: Automatic force map analysis) that (i) allows for a fast, high-throughput analysis of force mapping data, thus drastically speeding up data analysis; and (ii) generates a spatial map of the acquired force values. Using this system, we were able to reconstruct the image of SM/Chol Lo domains in a DOPC Ld phase based on the different breakthrough force values characterizing the two phases (Figure 2B,C). In addition, we could provide fast information about their thickness and consequently on the difference in height between the two phases (height mismatch) (Table 2). Generally, distinct force spectroscopy information about force or thickness values in two phase systems have been obtained by manually selecting micro-areas containing exclusively one or the other phase and performing force spectroscopy in such areas [27]. Although possible, this can turn again in time-consuming measurements. To overcome this issue, we exploited the simultaneous force and thickness information provided by force mapping to perform a cluster analysis on our samples (Figure 2D,E).

We have used this approach to follow the simultaneous changes in topography and mechanical properties of DOPC/SM/Chol (2:2:1) bilayers after addition of EqtII. With this approach, we could provide, in a very short time, a quantitative characterization of the membrane phases with and without the toxin. This processing tool, in addition, offers the unique advantage compared to separately imaging and performing force spectroscopy to unequivocally associate a specific force and thickness value to a region as small as 0.09 µm^2^ (sub-area of one membrane grid in our experiments) or less, depending on the size of the detected area. This allows, in principle, to differentiate between micro-areas that by simple imaging would look similar.

### 2.2. Actinoporins Affect the Topology of Lo Domains

**Figure 3:**
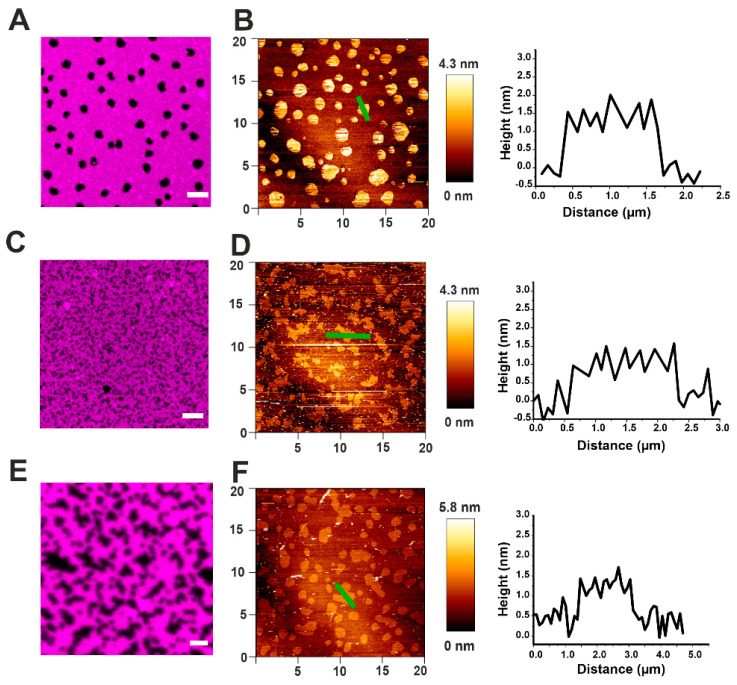
Effect of actinoporins on the topology of Lo domains. (**A**) Representative image of a DOPC/SM/Chol 2:2:1 membrane system presenting lipid phase separation by confocal microscopy. SM/Chol Lo domains are visualized like black round spots surrounded by the Ld-phase enriched in DOPC, labeled with DiD-C18 (magenta). (**B**) The same sample as in (**A**) imaged by AFM. Domains are visualized like brighter spots. The representative cross-section corresponds to the green line in the respective AFM image and shows the height mismatch between the two phases. (**C**–**F**) Confocal (**C**,**E**) and AFM (**D**,**F**) representative images of a DOPC/SM/Chol 2:2:1 system prepared from SUVs pre-incubated with EqtII (**C**,**D**) or FraC (**E**,**F**). Scale bar is 5 µm (**A**,**C**) and 2 µm (**E**). Images are representative of at least three independent experiments.

Supported lipid bilayers (SLB) containing Lo domains of SM/Chol in the DOPC Ld phase were imaged by AFM and compared with confocal microscopy (Figure 3). In confocal images, the domains appeared as dark spots, due to the exclusion of the lipid dye DID from ordered phases (Figure 3A), while by AFM they were displayed like brighter spots exceeding the surrounding Ld phase by around 1 nm in height (Figure 3B and Table 2). In both cases, Lo phases appeared in the form of round domains between 0.5–3 µm in diameter (Figure 3A,B). This characteristic round shape is due to the existence of line tension due to a height mismatch between the two lipid phases. Such a mismatch induces a tilt of the lipids and curvature stress to avoid the exposure of hydrophobic portions of the lipid tails to the water environment [28].

In order to see the effect of EqtII and FraC on the phase separation, we formed SLBs from small unilamellar vesicles (SUVs) pre-incubated with the toxin. In this scenario, the domains appeared smaller and lost their circular shape in favor of an irregular one (Figure 3C–F). However, the height mismatch with the Ld phase did not significantly change (see representative cross-section graphs in Figure 3B,D,F and Table 2).

**Figure 4:**
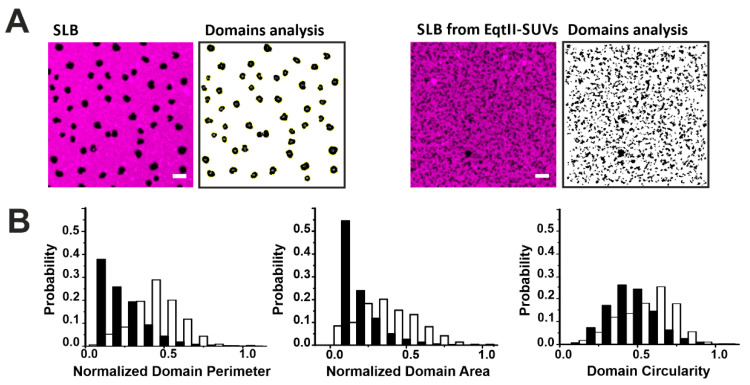
Characterization of Lo domains imaged by confocal microscopy in the presence of EqtII. (**A**) Confocal images of SLB from SUVs not incubated (left panels) or incubated (right panels) with EqtII. Confocal images have been treated with the particle analysis tool of Imagej that allows the estimation of the perimeter, area, and circularity of the domains. The scale bar is 5 µm. (**B**) Comparison of the perimeter, area, and circularity of the domains without (white bars) and with (black bars) the protein. Histograms are normalized for better comparison (the number of points varies between 600 and 6500 from at least two independent experiments).

To quantify these changes, we characterized the shape of the domains by their area, perimeter, and circularity in the presence or not of EqtII (Figure 4). The presence of the toxin drastically reduced the perimeter and area of the domains by 80% and 64%, respectively, while the circularity of the domains decreased by around 27% in all the analyzed samples (Figure 4B). It has been shown that increased line tension favors the formation of big circular domains to minimize the energetic cost associated to the domain boundary length [29,30,31,32,33,34]. Therefore, the reduction in the domain size and circularity is consistent with a reduction in the line tension at the phase boundaries induced by the toxin. This is in line with previous results obtained with other related actinoporins [16] and from other pore-forming proteins (PFPs) [35,36] and it may reflect a general mechanism of action for this family of proteins.

### 2.3. Automatic High-Throughput Characterization of the Mechanical Properties of DOPC/SM/Chol (2:2:1) Phases in the Presence of Actinoporins

**Figure 5:**
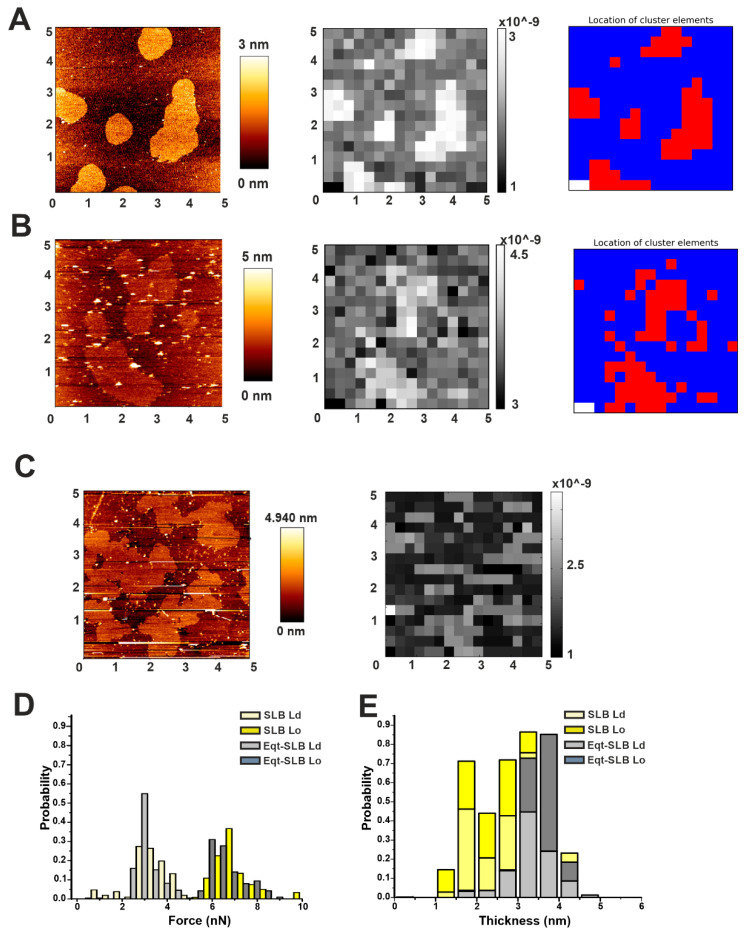
Characterization of Lo domains in the presence of actinoporins by automatic force mapping analysis. (**A**) (Left) Representative AFM figure of the DOPC/SM/Chol 2:2:1 membrane system prepared from SUVs, presenting Lo domains (brighter areas) whose thickness exceeds the surrounding Ld phase (darker area). (Centre) Force-map reconstructed image by our software. (Right) Map of the same 5 μm × 5 μm area with each spot colored according to its affiliation with one cluster determined by the KMeans algorithm. (**B**) Same as in (**A**) for samples prepared from SUVs pre-incubated with FraC. (**C**) Same as in the left and central panels of (**B**) for samples prepared from SUVs pre-incubated with EqtII. All images are representative of at least three independent experiments. (**D**,**E**) Comparison of force (**D**) and thickness (**E**) values of SLBs without (light and dark yellow bars) and in the presence (light and dark gray bars) of the protein. Histograms are normalized for better comparison (the number of points varied from 220 to 450 from at least two independent experiments).

To see if the changes in the morphology were associated to a change in the membrane properties of Lo domains, we performed force mapping on SLBs formed by SUVs pre-incubated with EqtII or FraC, and reconstructed the images by plotting the force values with our customized software (Figure 5). Even though we could observe relevant differences in the morphology compared to the control sample (Figure 5A–C), the irregular shape of the domains in the presence of the toxin could not be accurately reconstructed by plotting the force values with our spatial resolution (a sub-area in the grid is 0.09 µm^2^) (Figure 5B,C). Using the clustering tool provided with our software, we could plot the force distribution for the Lo and Ld clusters in the presence or not of EqtII. Apparently, the overall force distribution did not differ from the control sample (Figure 5D). Likewise, we plotted the thickness distribution based on the cluster separation between Lo and Ld phases (Figure 5E and Table 2). By simply plotting the thickness values, however, we could not distinguish the Ld and Lo populations, due to the overlapping of thickness distributions, having the DOPC/SM/Chol system a characteristic mismatch of less than 1 nm (Table 2 and [29,35,36]). In addition, from the cluster analysis, we calculated the maximum mismatch (considering that the average mismatch is not informative due to the overlap of thickness distribution between the Lo and Ld phase). These values, which agree with the average mismatch measured from the AFM images, were not significantly affected by the presence of EqtII (Table 2). Despite these data being statistically not significant, we could observe a propensity of the membrane to go more into a fluid phase at the expense of the Lo one (54% of the cluster points against 45% in the control sample, Table 2). Taken together, our results suggest that actinoporins may act locally at the interface between the two domains, not affecting the bulk domains.

**Figure 6:**
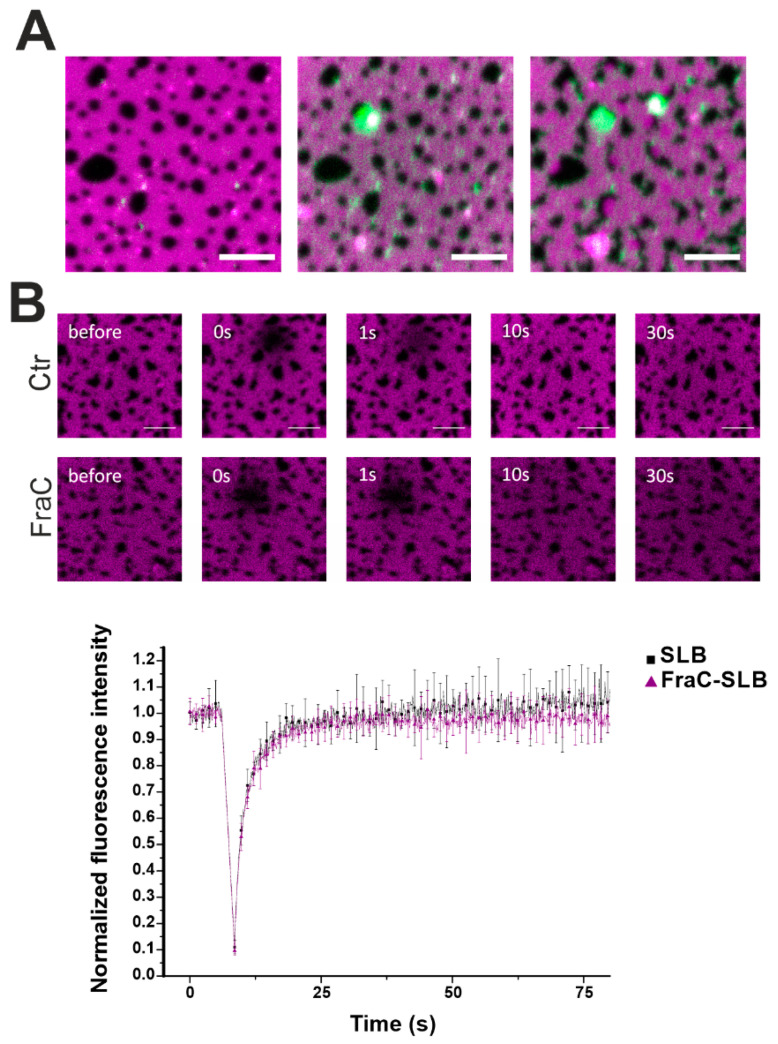
Confocal microscopy of DOPC/SM/Chol phases in the presence of FraC-488. (**A**) The Ld phase was stained with 0.1% DID (magenta) and FraCR126C was labelled with Alexa 488 (green). Picture taken immediately (left), 2 min (centre), and 15 min (right) after the addition of the protein. Scale bar is 5 µm. (**B**) Photobleaching experiments in the Ld phase without (black squares) and with (purple triangles) FraC. FraC does not alter the fluidity of the Ld phase. Dotted lines are for visual purposes only. Normalized fluorescence intensity over time is represented as the average ± SD over three (SLB) and six (FraC-SLB) repetitions.

To confirm our hypothesis on the action of actinoporins at the domain boundaries, we labeled EqtIICR126C or FraCR126C and performed confocal fluorescence microscopy (Figure 6). Interestingly, the toxin distributed initially into the Ld phase to cluster over time at the interface with the Lo domains (Figure 6A). This behavior is in line with findings by Rojko et al. showing that EqtII, upon binding to the membrane, concentrates at the domain boundaries [17]. Photobleaching measurements in the Ld phase showed that the overall fluidity of the Ld phase was not affected by FraC (Figure 6B).

In summary, these data suggest that, under our experimental conditions, actinoporins have a very local effect at the interface that could not be observed by ensemble information. Importantly, this conclusion could only be possible thanks to a direct visual correlation of the force values obtained by force mapping.

## 3. Discussion

In this study, we have developed the “Forcemap Analyser”, a fast, high-throughput and user-friendly analysis tool that generates a spatial map of the acquired force values. The reconstructed spatial force map can be directly visually compared to the topographical AFM image, thus allowing for a straightforward correlation of the structural and mechanical properties of the sample with a micro- to nano-meter resolution. This approach has important biological implications because it allows for a local investigation of the mechanical properties of lipid membranes, thus allowing for the detection of small membrane heterogeneities that would not be detected by ensemble measurements.

We have used this powerful tool to investigate the local perturbations induced by actinporins on the properties of liquid-ordered membrane phases.

The relevant changes in the domain morphology and size, as visualized by confocal and both AFM imaging and mapping, indicate a drastic membrane reorganization in the presence of the toxin. However, thanks to the ability of our software to provide separated information for Ld and Lo phases, we demonstrated that the mechanical properties of neither the bulk Ld nor the Lo phases were affected by the presence of the actinoporins. Altogether, these data strongly suggest that actinoporins induce alterations of the mechanical properties only locally at the phase boundaries. Fluorescence microscopy imaging showing the recruitment of actinoporins at the phase edges and FRAP experiments further corroborate this conclusion.

We hypothesize that these effects are associated with a lowering in the line tension associated with the suboptimal lipid packing at the domain boundary. In the context of the toroidal pore model proposed for these kinds of toxins [3], this behavior could imply a similar role in the stabilization of highly curved regions with suboptimal lipid-packing at the pore edges. The toxins would therefore behave as a line-actant, as previously documented for other PFPs [16,31]. This, in turn, means a local decrease of the force values at the edges of the domains, which justify why in the force representation obtained from the SLB in the presence of EqtII or FraC (Figure 5B,C) we could not clearly define and distinguish the Lo domains from the surrounding Ld phase.

However, SLBs showed an overall slight increase of 1.1 nm in the thickness if they were formed from SUVs pre-incubated with EqtII (Figure 5E and Table 2). This may also suggest the presence of the toxin in the Lo phase, although not clearly visible from our fluorescence images (Figure 6A), and we cannot exclude experimental artifacts.

Indeed, there are still ongoing debates on the preferential binding of EqtII, and in general for actinoporins, on ordered or disordered phases [13,18,19,20]. In fact, a study from Pedrera et al. questioned the relevance of segregated lateral phases for actinoporins’ action [20] and emphasized a more crucial role of the membrane curvature as a modulator of protein-partitioning in lipid phases [37]. It is most likely that actinoporins´ partition in pure lipid membranes with phase co-existence is strongly modulated by experimental conditions like the exact lipid composition, which modulates the different properties of Lo and Ld phases [13,14,17,19]. Moreover, SM properties like availability, fluidity, orientation, and dynamics are proposed to play a major role in actinoporins partitioning in the membrane [2]. Even in more complex systems, like membranes in living cells in which Lo macro-domains are not found, EqtII is able to recognize more dispersed, instead of clustered SM [18], and to oligomerize in spatially homogeneous toxin-induced cell blebs, while inducing the formation of microscopic domains enriched in typical raft components [15].

## 4. Conclusions

Force mapping by AFM is a powerful tool to combine topographical and mechanical information of biological samples. Nonetheless, due to the considerable amount of information provided, data processing can be time-consuming if performed manually. Here, we used an automatic tool for a reliable and fast analysis of force curves, simultaneously providing force and thickness information that are spatially plotted to reconstruct AFM images with additionally local quantitative mechanical information. This tool has allowed for the correlation of actinoporins preferential binding to phase boundaries with local changes in the mechanical properties that more likely occur at the membrane interfaces, resulting in a decrease of line tension. We propose that this line-actant activity of the protein is related to the stabilization of the highly curved membrane edge of toxin-induced pores. These findings could not be revealed by bulk analysis or ensemble force spectroscopy measurements. Furthermore, we could additionally get relevant information on the phase force, thickness, and in turn, on the phase mismatch, in a very short timescale. Our approach drastically speeds up the processing time of force mapping data and can be extended to any study where local variations of simultaneous topographical and mechanical information are relevant, such as those related to the mechanism of action of different PFTs.

## 5. Materials and Methods

### 5.1. Purification and Labelling of Equinatoxin II and Fragaceatoxin C

FraC, EqtII, and their single Cys mutant R126C were expressed in *E. coli* BL21-RIPL cells and purified by cation exchange (SP-Sepharose column, GE Healthcare, Uppsala, Sweden) and gel filtration (Superdex 200 HP column, GE Healthcare, Uppsala, Sweden) chromatography. EqtIIR126C was labelled with Alexa Fluor 488 maleimide (Invitrogen) (EqtII-488) according to the manufacturer’s instructions. The labelled protein was separated from the excess free dye by amicon ultra centrifugal filters with 10 kDa cut-off (Millipore, Darmstadt, Germany). Labelling efficiency was determined to be 86% and 87% for EqtIIR126C and FraCR126C, respectively, by fluorescence spectroscopy with a Specord S 100 (Analytik Jena, Jena, Germany). The activity of the labelled mutant was confirmed to be the same of the wild-type protein as previously reported [8].

### 5.2. Sample Preparation for Measurements in AFM

DOPC, egg SM, and Chol were purchased from Avanti Polar Lipids (Alabaster, AL). They were mixed in the following ratio: DOPC:SM:Chol (2:2:1 mol %, respectively). Planar supported lipid bilayers (SLB) were prepared as described in [27] and [38]. Briefly, after lipids preparation with the addition of 0.1% (mol/mol) 1,1′-dioctadecyl-3,3,3′,3′-tetramethylindodicarbocyanine 4-chlorobenzenesulfonate salt (DiD-C18) (Molecular Probes, Eugene, OR, USA), lipid mixtures were rehydrated in PBS buffer (2.7 mM KCl, 1.5 mM KH_2_PO_4_, 8 mM Na_2_HPO_4_, and 137 mM NaCl, pH 7.2) to a final concentration of 10 mg/mL and further diluted with SLB buffer (140 µL of 150 mM NaCl, 10 mM HEPES, pH 7.4) to a final concentration of 0.6 mg/mL. The suspension was then bath-sonicated to obtain small unilamellar vesicles (SUVs). The solution was then incubated in a water bath at 65 °C for 30 min, and after at room temperature for 30 min to induce the formation of lipid domains. Actinoporins were added to the SUV solution to a final concentration of 200 nM (approximate volume 2–3 µL) and incubated for 1 h at room temperature. The solution was placed in contact with a support preheated at 37 °C and made of freshly cleaved mica previously glued to a coverslip and incubated with CaCl_2_ 3 mM at 37 °C for 2 min. The samples were rinsed several times with SLB buffer and allowed to equilibrate at room temperature before analysis.

### 5.3. Confocal Microscope Imaging

SLBs were imaged using a commercial LSM 710 (Carl Zeiss, Jena, Germany) at 20 °C with a Zeiss C-Apochromat 40X, NA = 1.2 water immersion objective. Photobleaching experiments were conducted at 20 °C using the same microscope set-up described above. Control images were acquired before bleaching, then a 10 × 10 µm area was bleached at nominal 100% laser transmission, and a series of images (every 4 s) were captured immediately after bleaching. Domain size analysis was carried out by processing images with the “analyze particles” tool of ImageJ (http://imagej.nih.gov/ij/). Circularity was calculated as 4π (area/perimeter^2^) for every measured domain using ImageJ software.

### 5.4. AFM Imaging and Force Mapping Measurements

SLBs were imaged using a JPK NanoWizard II system (JPK Instruments, Berlin, Germany) mounted on an Axiovert 200 Inverted Microscope (Carl Zeiss) as described in [27]. Briefly, V-shaped silicon nitride cantilevers with a typical spring constant of 0.08 N/m were used to acquire intermittent contact (IC or tapping) mode images. The cantilever oscillation and the amplitude varied during the experiments by between 3–10 kHz and 0.3–0.6 V, respectively, to minimize the force of the tip on the bilayer. The scan rate was set to 0.7–1 Hz. The height, deflection and phase-shift signals were collected simultaneously in both trace and retrace directions.

Force spectroscopy measurements were acquired by the force map mode provided by the AFM. After imaging an area of the bilayer, a 5 µm × 5 µm region in the bilayer was selected. This area was divided by a 16 × 16 grid in 256 regions, resulting in 256 force curves. Before each force experiments, calibration of sensitivity, resonance frequency, and effective spring constant (via the thermal noise method) of the cantilever were performed. The total z-piezo displacement was set to 400 nm and the indenting speed was set to 800 nm/s and 200 nm/s for the approach and for the retraction, respectively. All experiments (for a minimum of three repetitions) were carried out at different positions of the bilayer under the same conditions, so that the effect of the speed of the breakthrough could be neglected. Force curves were processed by the JPK processing software. Accurate thickness and force measurements were obtained by applying a smoothing function, baseline correction and tip-sample separation correction to the force curves. All data were saved as a text file and further processed as described in the following section. Histograms of force and thickness values were obtained by plotting the data obtained by our customized software (see following section). Cluster analysis of thickness values were performed by the cluster analysis tool from Origin (OriginLab, Northampton, MA, USA) or from the cluster tool provided within our customized software.

### 5.5. Automatic Force Map Analysis

The text files from the force curves were automatically analysed by an in-house developed program called “Forcemap Analyser”. The program was written in python 2.7 (Python Software Foundation. Python Language Reference, version 2.7. Available at http://www.python.org), using the integrated development environment (IDE) *spyder* and the python modules *pandas* for internal data handling, *matplotlib* for the generation of graphical plots, *scikit-learn* for the KMeans clustering, and *wxPython* for the generation of the graphical user interface. The overall analysis workflow is described in Figure 1. Breakthrough forces were identified as kinks or points of locally high curvature in the force curve (Figure 1), which were detected using an algorithm for the detection of local maxima in arrays, written by Eli Billauer (http://www.billauer.co.il/peakdet.html, accessed on May 2013). The algorithm samples the curve and classifies maximal values in local neighbourhoods as peaks if they are surrounded by lower values. To also reliably identify those kinks that were not clearly defined and would not be considered real local maxima, we rotated the force curve in the coordinate system at angles of 0, 4.5, 9, 13.5, and 18 degrees. Kinks not corresponding to local maxima in the original curve are likely to appear as local maxima at a different rotation angle.

The force curve F(z) was represented by a Matrix $C^{N}$ with rows ${\left[F_{n},z_{n}\right]}_{n=1}^{N}$ where $F_{i}$ and $z_{i}$ are the force values and the corresponding z-positions of the AFM tip, respectively, and N denotes the total number of measurements. Rotation by angle φ was carried out as follows:

$$C_{\varphi }^{N}=C_{N}*\left(\begin{array}{cc} \cos\varphi & \sin\varphi \\ -\sin\varphi & \cos\varphi \end{array}\right).$$

We applied the peak detection algorithm to each rotated state $C_{\varphi }^{N}$ and provided in each case a set $S_{\varphi }$ of detected maxima represented by their coordinates ${\left[\hat{F_{i},}\hat{z_{i}}\right]}_{i=1}^{I_{\varphi }}$ with $I_{\varphi }$ being the number of detections at angle φ. Detections from all angles were then collected in the union set:

$$S=\underset{\varphi }{\cup }S_{\varphi }$$

The same kink might be detected at more than one rotation angle, so S usually contains redundant detections. Let $\left[\hat{F_{i},}\hat{z_{i}}\right]$ and $\left[\hat{F_{j},}\hat{z_{j}}\right]$, i≠j, be two detections in S. If the distance between them, ${\left({\left(F_{i}-F_{j}\right)}^{2}+{\left(z_{i}-z_{j}\right)}^{2}\right)}^{\frac{1}{2}}$, is smaller than a user-defined threshold, the detections are considered to originate from the same kink. In this case, only the detection from the smaller rotation angle is accepted because it is usually the most accurate one. This procedure was applied to S until no redundant detections were left.

In our force curves, every kink is followed by a kink with opposite curvature. Normally, the first kink corresponds to the encounter of the tip with the membrane and the second kink originates from the tip piercing and leaving the membrane. To find both kinds of kinks, we applied the detection algorithm to F(z) and −F(z). The *z*-distance between two corresponding kinks with opposite curvatures was considered as the height of the membrane.

We measured the piercing force on a 16 × 16 grid on the membrane with 0.3125 µm being the distance between two neighbouring measurement points (for a 5 × 5 µm image). Each of the resulting 256 force curves was analysed as described above and the piercing force and thickness values were determined. Thresholds were applied to these values to ensure that only plausible peaks were further analysed. The standard thresholds were 1.5 to 9 nm for the thickness values and at least 0.5 nN for the force values. If multiple peaks were detected above the threshold values, they were classified as primary and secondary, with the latter being closer to the mica support. Based on the collected values, we produced a 16 × 16 matrix of force values, which was visualized as in Figure 1. The image allowed for a quantitative analysis of spatial correlations between the force values. Black dots in the force map may correspond to no rupture event or a rupture event not detected by the software. Besides recreation of a 2D plot of the force values, the Forcemap Analyser program also allowed clustering of the force curves into two groups using the KMeans algorithm and the generation of plots and maps of these clusters. A summary of the overall features of the software are reported in Table 1. The automatic force mapping analysis tool is provided as a Python 2.7 executable file with a graphic user interface, and it is available at Github.

## Figures and Tables

**Table 1:** Features of the Forcemap Analyser program.

Reading of pre-processed data files;
Peak detection;
Application of user definable thresholds for force and threshold values to raw data;
Generation of force and thickness maps from raw and reloaded data;
Further options: marking of spots with missing or additional data, using the data of a secondary peak instead of the primary peak, setting the value range for the colour bar;
Calculation of simple peak statistics for the force curves;
Calculation of KMeans clusters and generation of plots and maps of these clusters;
Saving and reloading data;
Saving all pictures in different file formats (png, pdf and svg).

**Table 2:** Cluster analysis of DOPC/SM/Chol (2:2:1) phases in Supported Lipid Bilayers (SLB).

System	N° Points ^a^	Mean Thickness (nm)	Max Mismatch ^b^ (nm)	Average Mismatch from AFM Images
SLB	45% (Ld) ^c^ 55% (Lo)	2.2 ± 0.5 (Ld) 2.5 ± 0.6 (Lo)	1.2	1.0 ± 0.3
SLB from Eqt-SUV *	54% (Ld) 46% (Lo)	3.3 ± 0.5 (Ld) 3.6 ± 0.2 (Lo)	0.9	0.9 ± 0.2

^a^ Percentage of the number of points inside the cluster. ^b^ Maximum value of the difference between each Lo value and the mean Ld thickness values. ^c^ Ld is considered the cluster with lower, and Lo the one with higher force values. * SUV= small unilamellar vesicles
